# HOW DO INTERGENERATIONAL RELATIONSHIPS IMPACT THE HEALTH OF ASIAN AMERICAN OLDER ADULTS?

**DOI:** 10.1093/geroni/igac059.811

**Published:** 2022-12-20

**Authors:** Duy Nguyen, Yookyong Lee, Rui Liu

**Affiliations:** Sacred Heart University, Teaneck, New Jersey, United States; University of Alabama at Birmingham, Birmingham, Alabama, United States; Sacred Heart University, Fairfield, Connecticut, United States

## Abstract

Intergenerational relationships and filial piety are important values common to many Asian-ethnic groups. While a limited literature exists examining the health outcomes of older adults living with grandchildren in Asian countries, Asians in America have received less attention from researchers and policymakers. This study fills the knowledge gap by using data from the 2015-2019 American Community Surveys to examine the relationship between Asian ethnicity and living with grandchildren on health outcomes. This analysis focuses on respondents aged 65 and over from the six most populous Asian American groups: Chinese, Filipino, Indian, Japanese, Korean, and Vietnamese (n=100,538). Roughly half the sample lived with grandchildren. Weighted, adjusted logistic regression analyses tested for the effects of Asian ethnicity and living with grandchildren on 4 health outcomes: ambulatory, independent living, hearing, and vision difficulties. Multivariate analyses showed Filipino and Vietnamese older adults were more likely than Chinese to report difficulties across health outcomes. Additionally, individuals living with grandchildren were less likely to report ambulatory, independent living, and hearing difficulties. Joint effect analyses revealed Indian, Filipino, and Japanese Americans living with grandchildren reported more difficulties compared to the reference group. Overall, the results suggest that living with grandchildren can be a protective factor for the health outcomes of older Asian Americans, while having different impacts depending on ethnic origin. Future research needs to differentiate the impact of living with grandchildren across Asian ethnic groups. Further, culturally appropriate policy and practices are needed to promote successful aging among older Asian Americans living with grandchildren.

